# Nitrosative stress defences of the enterohepatic pathogenic bacterium *Helicobacter pullorum*

**DOI:** 10.1038/s41598-017-10375-1

**Published:** 2017-08-30

**Authors:** Margarida R. Parente, Elena Forte, Micol Falabella, Ivo G. Boneca, Miguel Teixeira, Alessandro Giuffrè, Lígia M. Saraiva

**Affiliations:** 1 Instituto de Tecnologia Química e Biológica António Xavier NOVA, Av. da República, 2780-157 Oeiras, Portugal; 2 Department of Biochemical Sciences, Sapienza University of Rome, Rome, Italy; 3 Institut Pasteur, Groupe Biologie et Génétique de la Paroi Bactérienne, Département de Microbiologie, Paris, France; 4 INSERM, Groupe Avenir, Paris, France; 5 CNR Institute of Molecular Biology and Pathology, Piazzale Aldo Moro 5, I-00185 Rome, Italy

## Abstract

*Helicobacter pullorum* is an avian bacterium that causes gastroenteritis, intestinal bowel and hepatobiliary diseases in humans. Although *H. pullorum* has been shown to activate the mammalian innate immunity with release of nitric oxide (NO), the proteins that afford protection against NO and reactive nitrogen species (RNS) remain unknown. Here several protein candidates of *H. pullorum*, namely a truncated (TrHb) and a single domain haemoglobin (SdHb), and three peroxiredoxin-like proteins (Prx1, Prx2 and Prx3) were investigated. We report that the two haemoglobin genes are induced by RNS, and that SdHb confers resistance to nitrosative stress both *in vitro* and in macrophages. For peroxiredoxins, the *prx2* and *prx3* expression is enhanced by peroxynitrite and hydrogen peroxide, respectively. Mutation of *prx1* does not alter the resistance to these stresses, while the single ∆*prx2* and double ∆*prx1*∆*prx2* mutants have decreased viability. To corroborate the physiological data, the biochemical analysis of the five recombinant enzymes was done, namely by stopped-flow spectrophotometry. It is shown that *H. pullorum* SdHb reacts with NO much more quickly than TrHb, and that the three Prxs react promptly with peroxynitrite, Prx3 displaying the highest reactivity. Altogether, the results unveil SdHb and Prx3 as major protective systems of *H. pullorum* against nitrosative stress.

## Introduction

*Helicobacter* (*H*.) *pullorum* is an enterohepatic species that colonizes the gastrointestinal tract of birds^1, 2^, mice and rats^3, 4^, and humans^1, 5^. The bacterium is recognised as a potential human pathogen, having been isolated from patients with digestive disorders and bacteraemia^5, 6^. *H pullorum* was reported to cause a pro-inflammatory response by inducing IL-8 through the NF-κβ pathway of epithelial cells^7^. Recently, we have shown that *H. pullorum* is internalised by and activates murine macrophages, stimulating the expression of pro-inflammatory cytokines such as TNF-α, IL-1β, IL-6 and murine MIP-2. Moreover, *H. pullorum* was shown to activate the mammalian inducible nitric oxide synthase (iNOS) that releases nitric oxide (NO)^8^.

NO and its derivatives, known as reactive nitrogen species (RNS), are well-established antimicrobials produced by the mammalian innate immune system. However, microbes attempt to avoid the deleterious effects of RNS through the action of specific detoxifying enzymes.

In bacteria, two major families of enzymes are primarily involved in NO detoxification, namely the flavodiiron NO reductases and flavohaemoglobins^9^. Flavohaemoglobins are bacterial globins, a diversified group of haem-containing globin-like proteins that also includes the single domain haemoglobins (SdHb) and truncated haemoglobins (TrHb)^10^. Flavohaemoglobins present a two-domain structure composed by an N-terminal haem-containing globin-like domain fused to a C-terminal reductase domain that binds NADH and FAD. SdHbs are single domain proteins that lack the reductase region and are able to perform NO detoxification, as shown for *Vitreoscilla* (*V*.) *stercoraria* Vhb and *Campylobacter* (*C*.) *jejuni* Cgb. A chimeric protein comprising *V. stercoraria* Vhb fused to the flavoreductase domain of *Ralstonia (R.) eutropha* flavohaemoglobin was reported to detoxify NO and to protect the heterologous host *Escherichia* (*E*.) *coli* against nitrosative stress^11^. The *C. jejuni cgb* mutant strain showed marked inhibition of aerobic respiration and hypersensitivity to NO and other nitrosating agents^12^. TrHbs are proteins shorter than SdHbs by 20–40 residues and with a characteristic 2-on-2 α-helical fold, and have been proposed to participate in oxygen and NO metabolisms^13^. For example, *Mycobacterium* (*M*.) *tuberculosis* truncated haemoglobin (HbN) exhibits NO dioxygenase activity and enhances the survival of *Salmonella* (*S*.) *enterica typhimurium* inside macrophages^14^. In *M. bovis*, deletion of the *hbn* gene generated a strain with impaired respiration and NO metabolizing capacity^15^. However, a limited protective effect against NO was described for *M. smegmatis* HbN^16^. *M. leprae* truncated haemoglobin (HbO), the only Hb apparently encoded in this bacterium was also shown to scavenge NO, hydrogen peroxide and peroxynitrite, been the later a toxic species generated by the reaction of NO with superoxide anion^17^. Finally, *Pseudoalteromonas haloplanktis* HbO catalyses peroxynitrite isomerization *in vitro*, and guards the bacterium from oxidative and nitrosative stresses^18^. The truncated haemoglobins of *C. jejuni* (Ctb) and *H. hepaticus* were characterised too^12, 19^, but the contribution to NO protection was, so far, only investigated for Ctb. Although *C. jejuni ctb* was induced by nitrosative stress, the protein did not confer NO protection^12^.

Bacterial peroxiredoxins (Prxs) are a group of ubiquitous peroxidases that detoxify a wide range of organic hydroperoxides, which are also implicated in protection against nitroxidative stress generated by peroxynitrite. Prxs are divided in six protein subfamilies based on amino acid sequence similarity, of which the alkyl hydroperoxide-reductase (AhpC)/Prx1, thiol peroxidase (Tpx) and bacterioferritin comigratory protein (BCP)/PrxQ are the most studied subfamilies^20^. Peroxiredoxins exhibit in their active site a strictly conserved catalytic cysteine residue, referred as the peroxidatic cysteine (C_P_), that is oxidised to sulfenic acid by the peroxide substrate. Some peroxiredoxins have another conserved cysteine residue outside the catalytic centre, termed resolving cysteine (C_R_), which forms a disulfide bond with C_P_ during the catalytic cycle. According to the number of catalytic cysteines, Prxs are classified as ‘2-Cys Prxs’, ‘atypical 2-Cys Prxs’, and ‘1-Cys Prxs’. Besides the catalytic cysteine(s), some peroxiredoxins contain other non-conserved cysteine residues that apparently are not involved in catalysis, such as*. S. aureus* AhpC, *C. jejuni* and *E. coli* Tpx^21^.

In general, bacterial peroxiredoxin-encoding genes are highly diverse in terms of transcriptional response and phenotype behaviour, and the proteins are usually considered as protective systems against oxidative stress. However, peroxiredoxins have also been reported to play a role in the detoxification of RNS, with *ahpC* mutants of *Listeria monocytogenes* ^22^, *Francisella tularensis* ^23^, *Brucella abortus* ^24^, *M. tuberculosis*, and *M. smegmatis* ^25^ showing increased sensitivity to peroxynitrite. Also, *H. pylori* AhpC reduces peroxynitrite to nitrite and is proposed to contribute to *H. pylori* resistance to RNS killing^26^.

Despite the increasing evidence in favour of the pathogenicity of *H. pullorum* to humans, the protein systems used by the bacterium to detoxify the chemical species generated by host defences remain unknown^27^. Therefore, we have searched the genome of *H*. pullorum for homologs of gene products putatively involved in RNS detoxification and analysed the role of these proteins in nitrosative stress protection.

## Results

### Analysis of the *H. pullorum* genome

Analysis of the available *H. pullorum* MIT 98–5489 genome retrieved no homologs of the major NO-detoxifying enzymes, i.e. of flavohaemoglobin and flavodiiron proteins^9, 28^. However, it revealed the presence of two putative Hb-like proteins, HPMG_00954 (also designated as HPMG_RS04780) and HPMG_00979 (HPMG_RS03725), as judged by their notable amino acid sequence identity (I) and similarity (S) with other bacterial Hb proteins (Supplementary Figure S1, Supplementary Information). The coding region of HPMG_00954 shares 56% I (74% S) and 50% I (69% S) with the single domain haemoglobins *C. jejuni* Cgb and *V. stercoraria* Vhb respectively, which are the best characterised single domain haemoglobins among bacteria. Furthermore, HPMG_00954 shares 42–48% I and 60–67% S with the globin domain of the flavohaemoglobins from *Staphylococcus* (*S*.) *aureus*, *Pseudomonas* (*P*.) *aeruginosa* and *E. coli*. One of the conserved residues of HPMG_00954 is the haem-binding histidine residue located in helix F of all known haemoglobins^13, 29^. Therefore, we will refer to HPMG_00954 as the single domain haemoglobin (SdHb) of *H. pullorum*.

The *H. pullorum* HPMG_00979 gene product was instead designated as a truncated haemoglobin (TrHb), because it shares significant amino acid sequence identity and similarity with several previously studied bacterial truncated haemoglobins, including *C. jejuni* Ctb (64% I; 78% S), *H. hepaticus* HbP (66% I, 82% S), *M. tuberculosis* HbN (29% I; 39% S), *M. tuberculosis* HbO (17% I; 35% S) and *P. haloplanktis* HbO (21% I, 45% S) (Supplementary Figure S1B).

In addition, three peroxiredoxin-like proteins were identified in *H. pullorum*. These proteins are: HPMG_00817 (HPMG_RS04115, named Prx1), HPMG_00739 (HPMG_RS03725, named Prx2), and HPMG_00529 (HPMG_RS02600, named Prx3), which according to the amino acid sequence comparison belong, respectively, to the Bcp/PrxQ, AhpC/Prx1 and Tpx families. *H. pullorum* Prx1 shares significant amino acid sequence identity and similarity with Bcp of *C. jejuni* (61% I, 72% S) and *H. pylori* (52% I, 77% S). *H. pullorum* Prx2 is a homolog of AhpCs of *C. jejuni* (69% I, 60% S) and *H. pylori* (46% I, 66% S), while *H. pullorum* Prx3 shares similarity with Tpxs from *C. jejuni* (83% I, 72% S) and *H. pylori* (87% I, 66% S) (Supplementary Figure S2).

*H. pullorum* Prx1 contains two conserved cysteine residues at positions 45 and 50, corresponding to putative C_P_ and C_R_, respectively. Also, *H. pullorum* Prx2 has two conserved cysteines, at positions 49 (putative C_P_) and 169 (putative C_R_), and a non-conserved cysteine at position 156, and *H. pullorum* Prx3 has two conserved cysteines at positions 58 (putative C_P_) and 92 (putative C_R_) (Supplementary Figure S2). Members of the peroxiredoxin protein family usually contain highly conserved proline, threonine and arginine residues in their catalytic centre^21^, and these residues are also present in the primary sequences of the *H. pullorum* peroxiredoxins (Supplementary Figure S2).

### Nitrosative stress induces the expression of *sdHb* and *prx2*

The transcription of *H. pullorum sdHb*, *trHb* and *prx*1-3 genes was analysed by quantitative real-time RT-PCR using total RNA extracted from *H. pullorum* cells exposed to chemical stresses.

**Figure 1:**
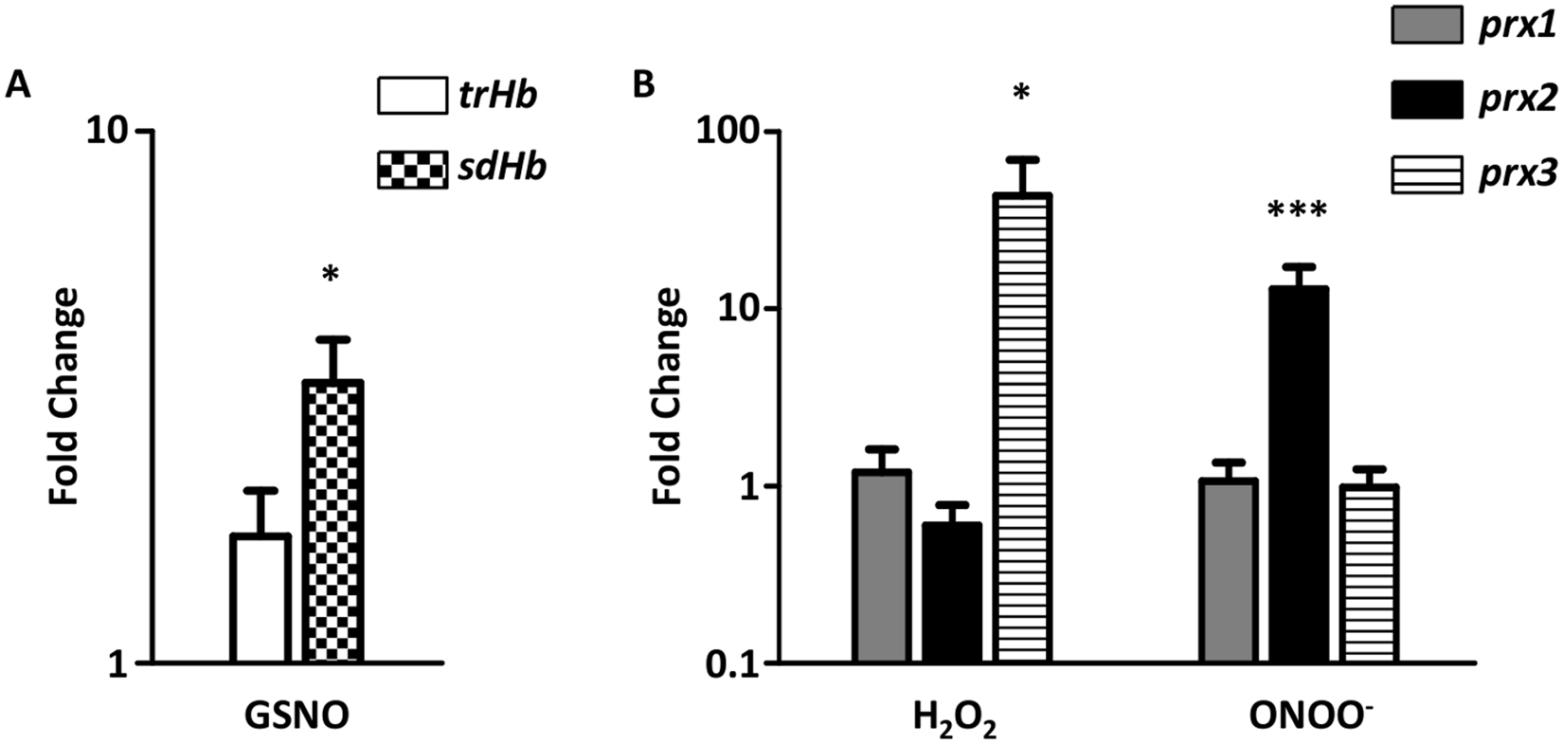
Transcription of haemoglobin and peroxiredoxin genes in *H. pullorum* stressed cells. (**A**) Expression of *trHb* (white bar) and *sdHb* (chess bar) in *H. pullorum* cells exposed to GSNO (100 µM). (**B**) Expression of *prx1* (dark grey bar), *prx2* (black bar), and *prx3* (stripped bar) in *H. pullorum* cells exposed to hydrogen peroxide (H_2_O_2_, 50 µM) or peroxynitrite (ONOO^-^, 50 µM). Total RNA was collected after 60 min of exposure of cells to GSNO (**A**), 30 min exposure to hydrogen peroxide and 15 min exposure to peroxynitrite (**B**). Fold change values represent the expression level ratio between treated and untreated cultures. At least two biological samples were analysed in duplicate. Data represent mean ± standard error with *t*-test. *P < 0.05, ***P < 0.0001.

Exposure of cells to the nitrosative stress inducer S-nitrosoglutathione (GSNO, 100 µM, 60 min) increased only slightly the transcription level of *trHb* (1.7 ± 0.6 fold), had a significant effect on the *sdHb* expression (3.4 ± 1.3) (Fig. 1A), and caused no major changes in the transcription of any of the peroxiredoxins genes (data not shown).

The transcription of peroxiredoxins was also analysed in cells exposed to either the oxidative agent hydrogen peroxide (H_2_O_2_, 50 µM, 30 min), or peroxynitrite (ONOO^−^, 50 µM, 15 min) which is a strong oxidant formed by the reaction of NO with superoxide anion. Hydrogen peroxide markedly enhanced the transcription of *prx3*, but not of the other two peroxiredoxins. Peroxynitrite significantly increased the expression of *prx2*, but did not modify the transcription of *prx1* and *prx3* (Fig. 1B).

These results point to a role of SdHb and Prx2 in the defence of *H. pullorum* against nitrosative and oxidative stresses, respectively. Therefore, we further evaluated the contribution of these gene products to the stress resistance of *H. pullorum* by producing single and combined deletion mutants of the haemoglobins and Prx-like genes, and analysing their phenotypes.

### SdHb protects *H. pullorum* from nitrosative stress

Single and double gene deletion-insertion mutants of *H. pullorum sdHb*, *trHb* and *prx*1-3 genes were constructed by homologous recombination using the primers described in Methods. Although the *H. pullorum* ∆*trHb*, ∆*sdHb*, ∆*trHb*∆*sdHb*, ∆*prx1*, ∆p*rx2* and ∆*prx1*∆*prx2* mutant strains were successfully obtained, the construction of the ∆*prx3* mutant was not achieved in spite of several attempts. When cultured under standard conditions, most mutants grew as well as the wild type, except *∆prx2* and ∆*prx1*∆prx2 that exhibited significant growth impairment (Supplementary Figure S3), therefore preventing further studies.

**Figure 2:**
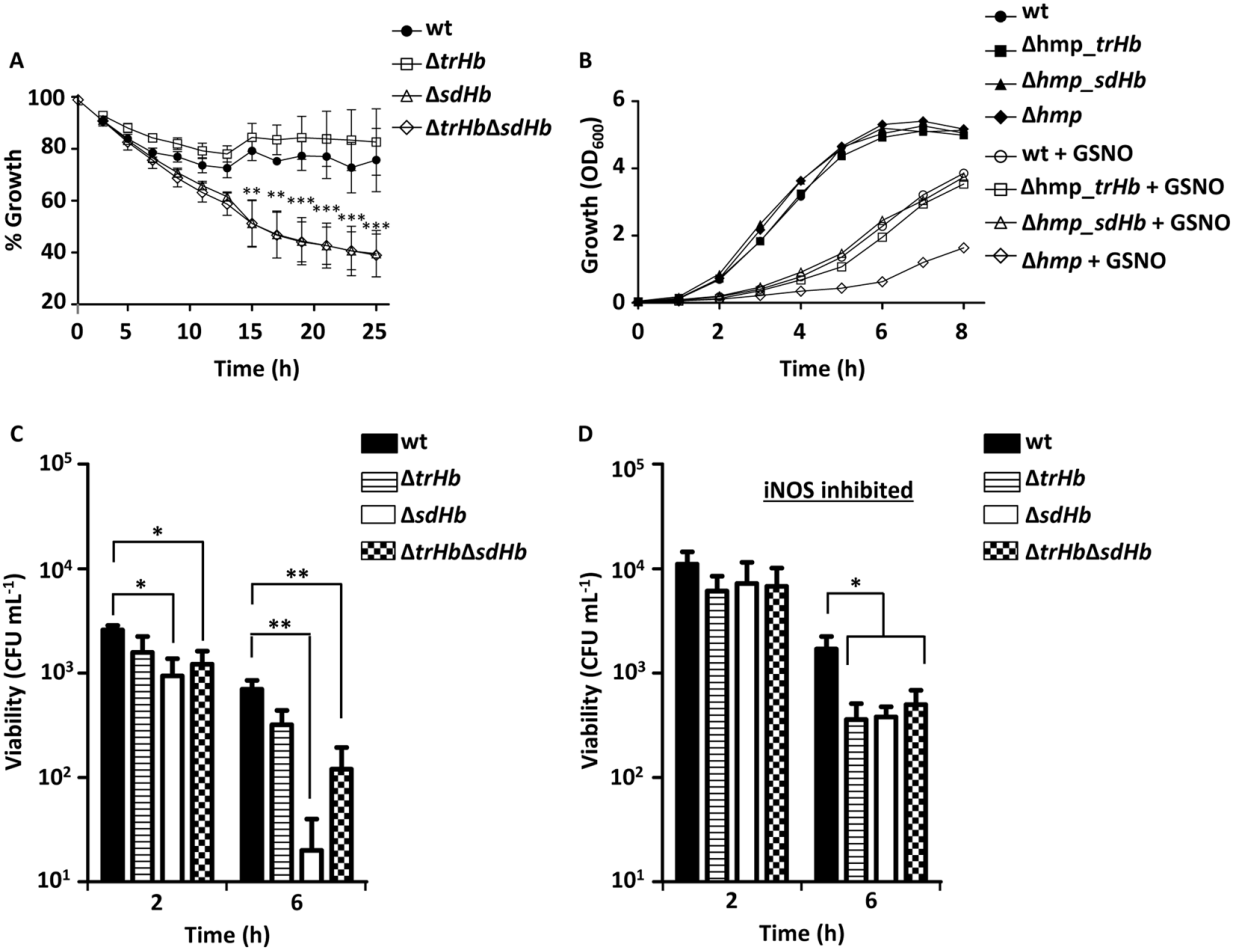
*Helicobacter. pullorum* haemoglobin protects against nitrosative stress. (**A**) Growth of *H. pullorum* wild type (filled circles), ∆*trHb* mutant (open squares), ∆*sdHb* mutant (open triangles), and ∆*trHb*∆*sdHb* double mutant (open diamond) when exposed to 100 µM GSNO. Growth percentage represents the ratio of the OD_600_ measured for stress-exposed versus untreated cells, at the indicated times. Four independent experiments were done, and data represent the mean ± standard error, with two-way ANOVA and Bonferroni test. **P < 0.01, ***P < 0.0001. (**B**) Growth curves of *E. coli* ∆*hmp* mutant strain complemented with *H. pullorum* TrHb or SdHb and exposed to nitrosative stress. Growth of *E. coli* wild type and ∆*hmp* mutant strains transformed with the empty vector pFLAG (circles and diamonds, respectively), and of *E. coli ∆hmp* expressing either *H. pullorum* TrHb (squares) or SdHb (triangles), in the absence (filled symbols) or presence (open symbols) of 200 µM GSNO. (**C**) Activated macrophages J774A.1 and (**D**) treated with the iNOS inhibitor L-NMMA were infected with *H. pullorum* wild type (black bar), ∆*trHb* (striped bar), ∆*sdHb* (white bar) and ∆*trHb*∆*sdHb* (chess bar), at a MOI of 100. Viable counts were determined after 2 and 6 h of infection. At least two independent experiments were analysed in duplicate. Data represent the mean ± standard error with *t-*test. *P < 0.05, **P < 0.01.

The ∆*trHb*, ∆*sdHb* and ∆*trHb*∆*sdHb* mutants were grown to the exponential phase and exposed to 100 µM GSNO. The ∆*trHb* mutant was as resistant to GSNO as the parental strain (Fig. 2A), while ∆*sdHb* and the double ∆*trHb*∆*sdHb* mutant both displayed comparably higher sensitivity (Fig. 2A).

Complementation assays were also conducted to test the ability of *H. pullorum* TrHb and SdHb proteins to rescue the deficient growth of the *E. coli* flavohaemoglobin deletion mutant (*∆hmp*) under nitrosative stress. These studies were done in *E. coli* as no plasmid for complementation in *H. pullorum* is so far available. Therefore, the *E. coli ∆hmp* mutant was transformed with plasmid pFLAG expressing either TrHb or SdHb and the resistance to GSNO (200 µM) was tested. As shown in Fig. 2B, over-expression of *H. pullorum* SdHb and TrHb markedly enhanced the resistance of the *E. coli ∆hmp* strain to GSNO (Fig. 2B).

Concerning *H. pullorum* peroxiredoxin 1, the resistance of the *∆prx1* mutant to nitrosative and oxidative stress was analysed by exposing cells to hydrogen peroxide (1 or 5 mM) or peroxynitrite (50 µM). In all cases, the mutant showed no increased sensitivity when compared with the wild type (Supplementary Figure S4).

### SdHb protects *H. pullorum* upon macrophages infection

The contribution of haemoglobins to *H. pullorum* survival within macrophages was assessed by incubation of wild type, ∆*trHb*, ∆*sdHb* and ∆*trHb*∆*sdHb* mutant strains in murine activated macrophages J774A.1. After 2 h of infection, there was no significant difference between the survival of *H. pullorum* wild type and ∆*trHb* while the viability of the ∆*sdHb* and ∆*trHb*∆*sdHb* mutants was lower. After 6 h of infection, the viability of the ∆*sdHb* and ∆*trHb*∆*sdHb* mutant strains became significantly lower than that of the wild type (Fig. 2C). Furthermore, ∆*sdHb* and ∆*trHb*∆*sdHb* mutants recovered viability when macrophages were treated with L-NAME (Fig. 2D and Supplementary Figure S5), a compound that inhibits the production of NO by the inducible nitric oxide synthase (iNOS) of mammalian cells^30^.

The ability of peroxiredoxin 1 to promote the survival of *H. pullorum* in murine macrophages was also analysed by incubation of the ∆*prx1* mutant with activated macrophages. The results showed that the behaviour of the ∆*prx1* mutant strain does not differ from that of the wild type (Supplementary Figure S4D).

### Reactivity of *H. pullorum* haemoglobins and peroxiredoxins

To further evaluate and to distinguish the role of these RNS protective systems, all proteins were recombinantly produced, purified and biochemically characterised. Expression in *E. coli* of *H. pullorum* yielded stable proteins with apparent molecular masses that are in agreement with those predicted from the gene products fused to His-tags, plus one haem group in the case of globins: 19.2 KDa for SdHb; 18.5 KDa for TrHb; 19.7 KDa for Prx1; 24.2 KDa for Prx2; and 19.4 KDa for Prx3.

**Figure 3:**
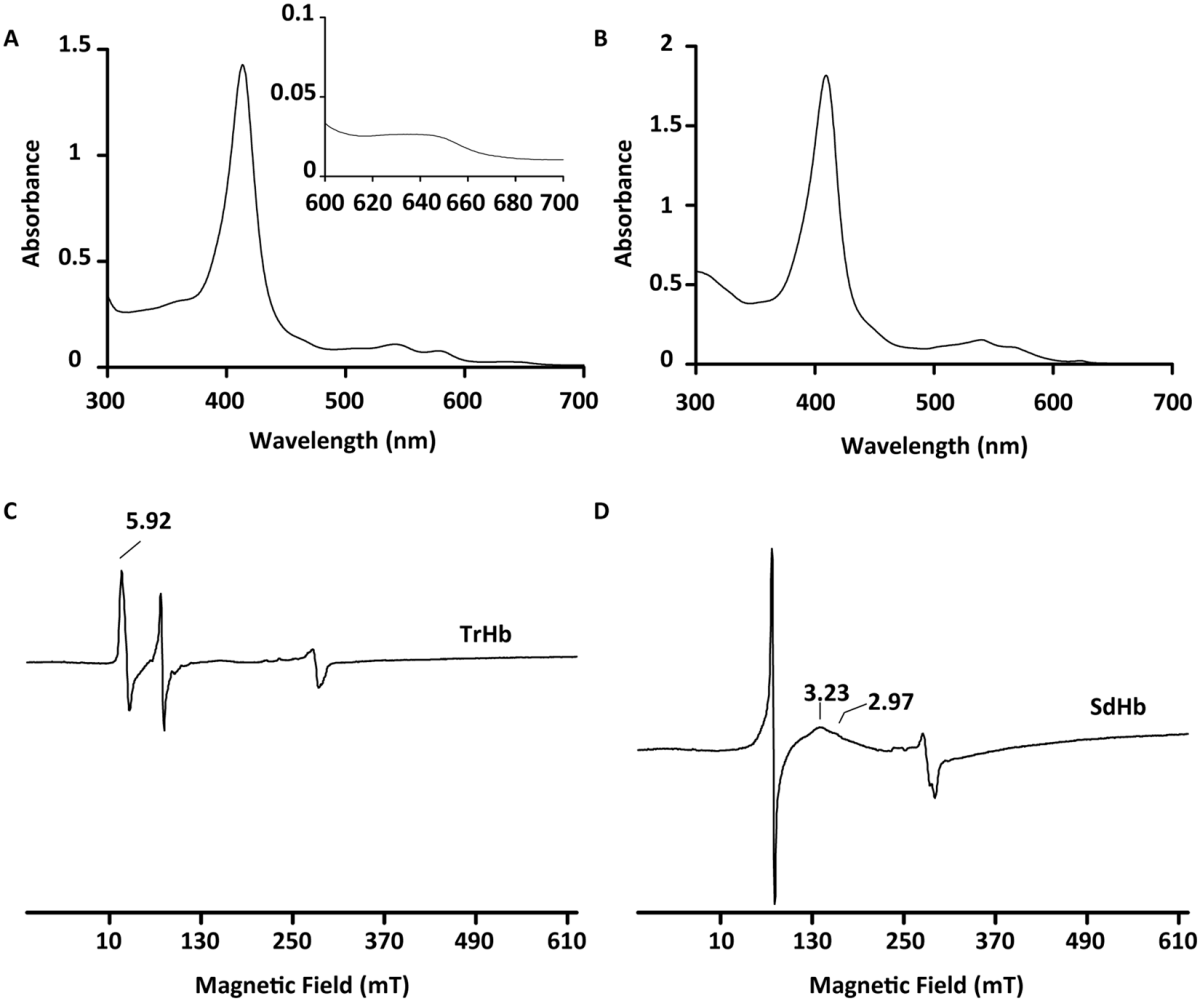
Spectroscopic properties of *H. pullorum* haemoglobins. UV-visible absorption spectra of as isolated (oxidised) *H. pullorum* TrHb (**A**) (Inset shows the 600–700 nm region expanded) and SdHb (**B**). EPR spectra of TrHb (**C**) and SdHb (**D**) at 12 K, 9.4 GHz. The peak at g = 4.3 derives from a cavity impurity.

As isolated, TrHb and SdHb presented spectroscopic features typical of haem-containing proteins with Soret absorption bands centred at 414 and 410 nm, respectively (Fig. 3A and B). By pyridine haemochrome assay, the haem *b* content was found to be ~0.7 per globin, a value consistent with the presence of one haem group per molecule. The two globins in the deoxy, oxy and met state displayed characteristic optical features (absorbance maxima and estimated extinction coefficients reported in Supplementary Table S1).

*H. pullorum* haemoglobins were also investigated by EPR spectroscopy. The spectrum of TrHb presented resonances characteristic of a high-spin ferric haem, with g_max_ = 5.92 (Fig. 3C). Accordingly, the UV-visible absorption spectrum of TrHb exhibited a broad charge transfer band with a maximum at ca 638 nm, which is a mark of ferric high-spin haem containing proteins (Fig. 3A). The EPR spectrum of *H. pullorum* SdHb exhibited only a broad resonance with a maximum at 3.2, indicative of a low-spin ferric haem containing protein, and resonances in the low-magnetic field region were not observed (Fig. 3D).

Next, the ability of *H. pullorum* Hbs to react with NO as well as the reactivity of the *H. pullorum* Prxs with peroxynitrite were assayed by time-resolved absorption spectroscopy, performing stopped-flow experiments on the isolated recombinant proteins.

### Reactivity of *H. pullorum* haemoglobins with nitric oxide

**Figure 4:**
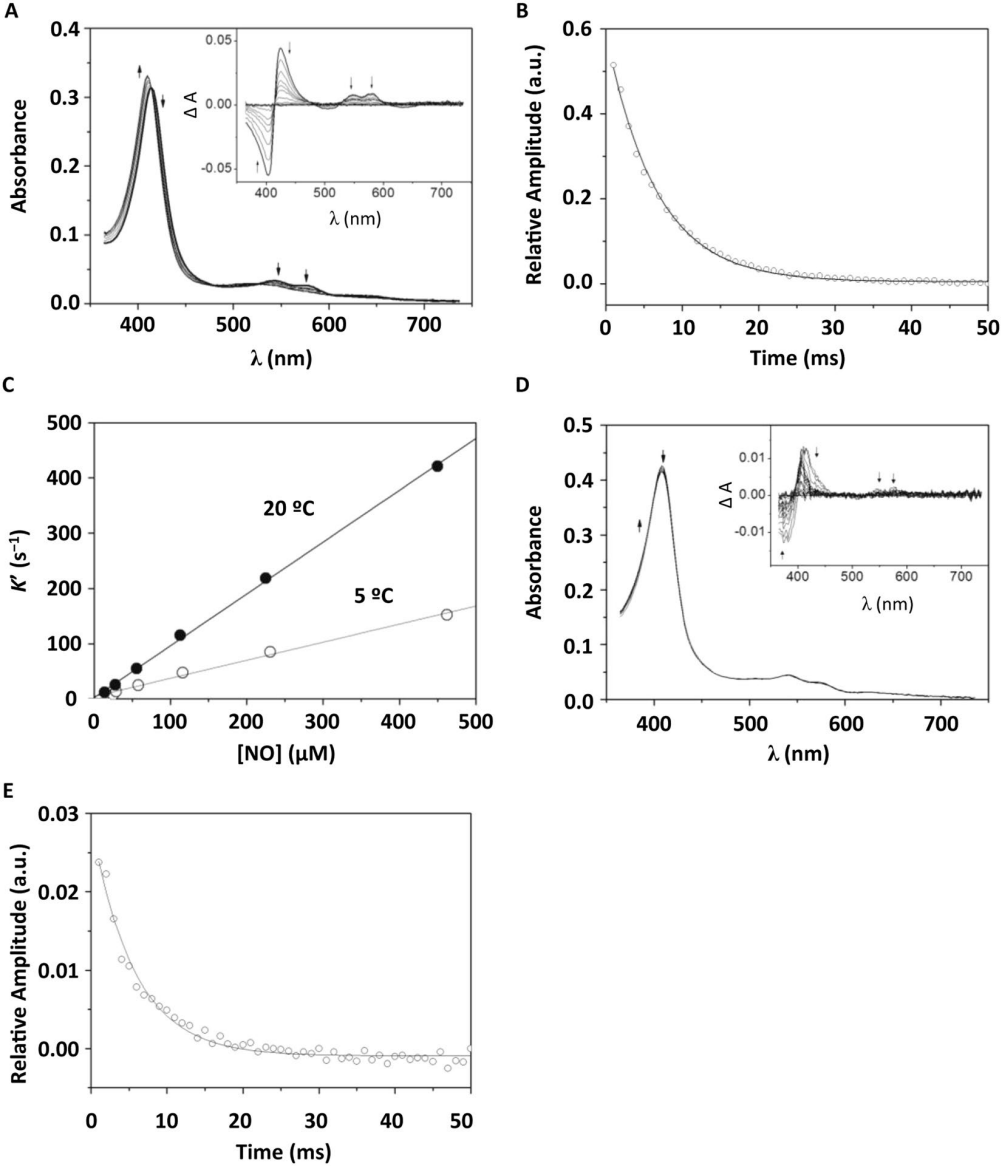
Reaction of *H. pullorum* TrHb and SdHb with NO. (**A**) Absorption spectra of TrHb collected over 50 ms at 5 °C after mixing 5.2 μM oxygenated TrHb with 925 μM NO and corresponding spectral changes (inset). (**B**) Reaction time course as obtained by global fit analysis of the data in panel A and its best fit to a single exponential (*k*′ = 152 ± 5 s^−1^). (**C**) For TrHb, the panel depicts the [NO] dependence of the observed rate constant measured at 5 °C and 20 °C and corresponding second order rate constants (*k* = 3.3 ± 0.1 × 10^5^ M^−1^ s^−1^ and *k* = 9.4 ± 0.1 × 10^5^ M^−1^ s^−1^) obtained by data regression analysis. (**D**) Absorption spectra of SdHb collected over 50 ms at 5 °C after mixing 11.2 μM oxygenated SdHb with 900 nM NO and corresponding spectral changes (inset). (**E**) For SdHb, reaction time course as measured at 414 nm–378 nm and its best fit to a single exponential (*k*′ = 203 ± 41 s^−1^).

Both *H. pullorum* globins (TrHb and SdHb) proved to react with NO, though at markedly different rates. The reactions were kinetically investigated by stopped flow spectroscopy, analysing the time-resolved absorption changes measured after rapidly mixing each protein in the ferrous oxygenated state with anaerobic solutions of NO. Prior to the experiments, formation of the ferrous oxygenated proteins was confirmed spectrophotometrically (Figure S6). A representative data set of the reaction of *H. pullorum* TrHb with NO is shown in Fig. 4. At 5 °C, the reaction of the protein (2.6 μM) with a large excess of NO (463 μM) occurs on a relatively short time scale (50 ms). By reacting with NO, the protein, initially in the ferrous oxygenated state, converts into the ferric state, as revealed by the observed absorption changes (Fig. 4A). Global fit analysis of such a data set showed that, under the tested experimental conditions, the reaction follows a single-exponential time course, best fitted with an observed rate constant *k*′ = 152 ± 5 s^−1^ (Fig. 4B). The second-order rate constant of the reaction was obtained at 5 °C (*k* = 3.3 ± 0.1 × 10^5^ M^−1^ s^−1^) and 20 °C (*k* = 9.4 ± 0.1 × 10^5^ M^−1^ s^−1^) by investigating the reaction at varied NO concentrations and fixed protein concentration (Fig. 4C).

More challenging was the kinetic investigation of the reaction of NO with *H. pullorum* SdHb, the reaction being much faster than that observed with TrHb. For this reason, and in order to be time-resolved by stopped-flow spectroscopy, the reaction with SdHb had to be investigated at low temperature (5 °C) and with relatively low concentrations of NO (<500 nM after mixing). In these assays, to fulfil pseudo-first order conditions, the protein was in >10 fold molar excess with respect to NO and, therefore, only a minor fraction of the protein was oxidized after mixing, thereby resulting in small absorption changes with unfavourable signal-to-noise ratio. The much faster reaction of SdHb, compared to TrHb, is documented in Fig. 4D,E. This representative data set shows that, despite the low temperature (5 °C) and the low NO concentration (450 nM after mixing), the NO-mediated oxidation of ferrous oxygenated SdHb (5.6 μM) is complete within 50 ms. Under the tested experimental conditions, the reaction proceeds at an observed rate constant *k*′ = 203 ± 41 s^−1^, consistent with a second order rate constant of 3.6 × 10^7^ M^−1^ s^−1^, two orders of magnitude greater than that measured for *H. pullorum* TrHb under the same conditions.

### Reactivity of reduced *H. pullorum* peroxiredoxins with peroxynitrite

**Figure 5:**
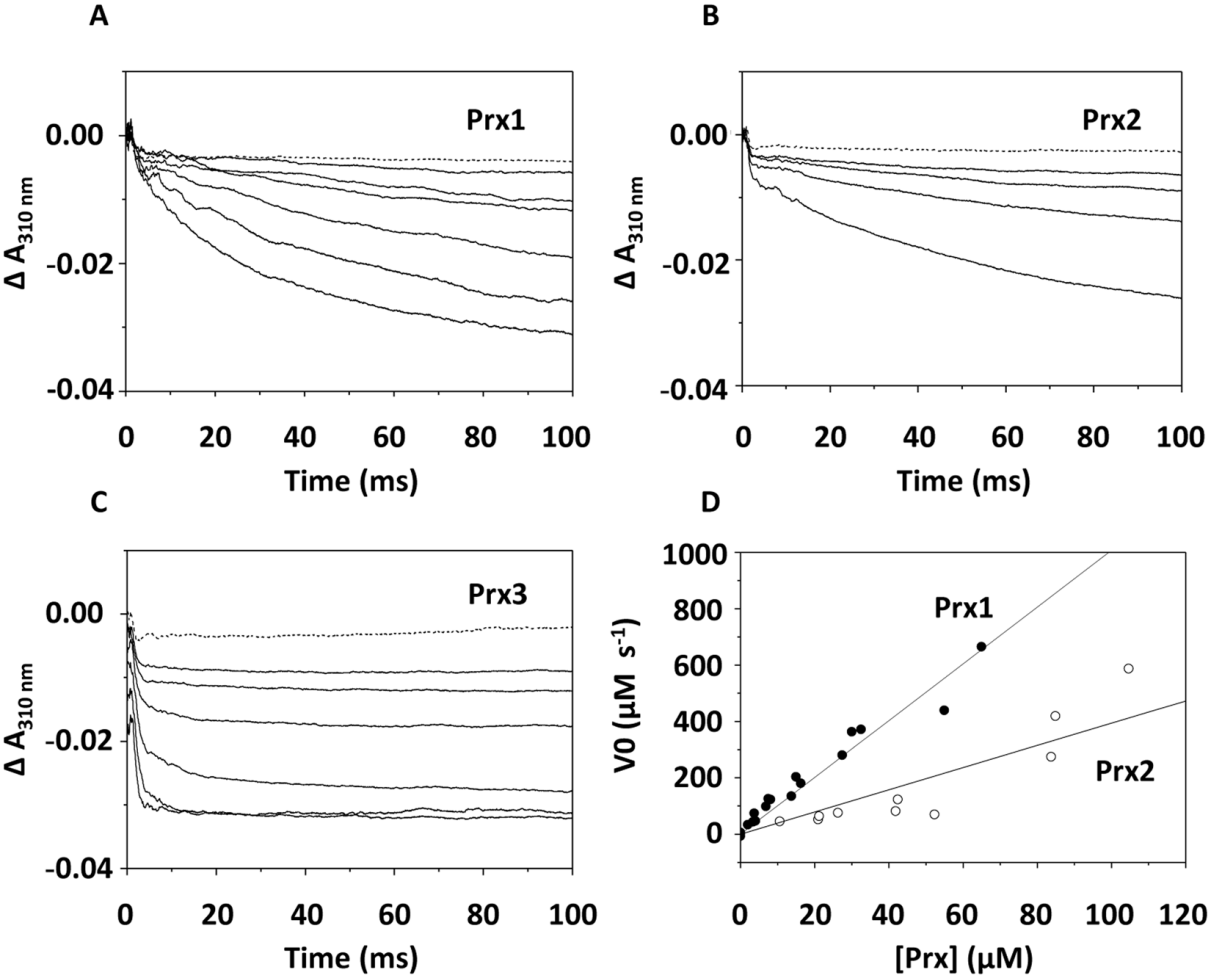
Reaction of *H. pullorum* reduced peroxiredoxins with peroxynitrite, and initial rate of peroxynitrite decomposition by Prx1 and Prx2. Absorption changes measured at 310 nm after anaerobically mixing peroxynitrite (ONOO^−^) with Prx1 (**A**), Prx2 (**B**) or Prx3 (**C**) at increasing concentrations. Temperature = 5 °C. Concentrations after mixing: (**A**) [Prx1] = 0–2.0–4.0–8.1–16.2–32.5–65.0 µM, [ONOO^−^] = 19 µM; (**B**) [Prx2] = 0–10.1–21.2–42.5–85.0 µM, [ONOO^−^] = 24 µM. (**C**) [Prx3] = 0–2.5–5.0–10.1–21.2–42.5–85.0 µM, [ONOO^−^] = 20 µM. (**D**) The rates were measured at 5 °C, at increasing concentrations of Prx1 (closed circles) and Prx2 (open circles). From linear regression analysis, the following second-order rate constants were estimated: *k* = 5.1 ± 0.8 × 10^5^ M^−1^ s^−1^ (Prx1) and *k* = 1.8 ± 0.3 × 10^5^ M^−1^ s^−1^ (Prx2).

Peroxynitrite was promptly degraded by the reduced proteins Prx1, Prx2 and Prx3 of *H. pullorum*. A representative data set collected at 5 °C is depicted in Fig. 5. The kinetic traces acquired at 310 nm clearly show that ONOO^−^ mixed with the buffer alone (100 mM phosphate buffer pH = 7.0 with 0.2 mM DTPA) is stable over the first 100 ms (dashed lines). A significantly faster decomposition is instead observed when ONOO^−^ is mixed with any of the three proteins in the reduced state (solid lines). At 5 °C, the reaction with Prx1 or Prx2 was sufficiently slow to be time-resolved by stopped-flow technique (Fig. 5A and B). In contrast, despite the low temperature, the reaction with Prx3 was much faster and a significant fraction of the reaction occurred over the first milliseconds, thus overlapping with a small artefactual signal invariantly observed immediately after mixing (Fig. 5C). Therefore, whereas the initial rates could be reliably measured for the reaction of ONOO^−^ with Prx1 and Prx2, such analysis could not be performed in the case of Prx3, the reaction being too fast. As a control, we confirmed that no reaction is observed over the same time scale (100 ms), when the *H. pullorum* proteins are pre-oxidised with an excess of hydrogen peroxide (~300 µM) prior to mixing with ONOO^−^ (not shown). In agreement with previous work^31^, in the case of Prx1 and Prx2 the initial rate of ONOO^−^ decomposition was found to be proportional to the protein concentration, the reaction with Prx1 being slightly faster than with Prx2 (5.1 ± 0.8 × 10^5^ M^−1^ s^−1^ vs 1.8 ± 0.3 × 10^5^ M^−1^ s^−1^, Fig. 5D). ONOO^−^ decomposition by reduced Prx3 was much faster than observed with Prx1 and Prx2, and the reaction time courses are compatible with a second-order rate constant *k* above 10^6^ M^−1^ s^−1^. The *k* values estimated for the proteins from *H. pullorum*, particularly for Prx1 and Prx2, are similar to those measured for *Giardia* Prxs at the same temperature^31^. The values fall within the range reported for Prxs from different microbial sources^32^, taking into account that the published values were obtained at higher temperatures (25 °C or 37 °C).

## Discussion

This work sheds light on the defence mechanisms against nitrosative stress in the bacterial pathogen *H*. pullorum. We report that the *H. pullorum* SdHb and TrHb encoding genes are induced by GSNO, particularly SdHb. Similarly, the homologous genes of *C. jejuni cgb* and *ctb* were reported to be up-regulated by nitrosative stress generators, such as GSNO, through the nitrosative stress-sensing regulator NssR^33^. In *H. pullorum* a similar regulation may exist as the genome codes for a protein sharing 49% identity and 68% similarity with *C. jejuni* NssR.

Phenotypic studies revealed that, when compared with the parental strain, the *H. pullorum* ∆*sdHb* mutant is more sensitive to nitrosative stress, whereas the ∆*trHb* mutant exhibits no major differences. Accordingly, the ∆*sdHb*∆*trHb* double mutant strain responded to GSNO as did the ∆*sdHb* strain. Similar results were obtained for *C. jejuni*, with the single-domain Hb mutant (∆*cgb*) showing decreased survival under nitrosative stress conditions and the viability of the truncated Hb mutant (∆*ctb*) did not differing from that of the wild type strain^33^.

Complementation of *E. coli ∆hmp* with either *H. pullorum* haemoglobins restored the resistance of the bacterium to nitrosative stress. Likewise, expression in the *E. coli* ∆*hmp* mutant of the truncated haemoglobins HbN of *M. tuberculosis* ^34^, HbO of *P. haloplanktis* ^18^, or the single domain haemoglobins Cgb of *C. jejuni* ^35^ and Vhb of *V. stercoraria* fused with the flavoreductase domain of *R. eutropha* ^11^ suppressed the growth impairment observed under nitrosative stress conditions. Furthermore, the HbN overexpression also conferred substantial protection to the *S. enterica Typhimurium hmp* mutant against RNS^14^. In contrast, the *C. jejuni* Ctb failed to restore the phenotype of the *E. coli* ∆*hmp* ^35^.

The NO protection provided by *H. pullorum* TrHb to the *E. coli ∆hmp* is most likely due to the overexpression of TrHb, while the lack of phenotype of the *H. pullorum trHb* mutant under stress conditions possibly reflects the lower activity of TrHb compared to that of SdHb.

So far, the available data on the protective role of bacterial haemoglobins during macrophage infection are very limited. Our *in vivo* studies revealed that SdHb improves the ability of *H. pullorum* to survive the nitrosative stress generated by macrophages, while TrHb does not. Interestingly, upon macrophage infection, the ∆*sdHb*∆*trHb H. pullorum* double mutant was not as sensitive as the ∆*sdHb* mutant, possibly due to the up-regulation in the double mutant of other *H. pullorum* defence systems. Previous studies in *M. tuberculosis* showed that overexpression of the truncated globin HbN augmented the survival of the pathogen within THP-1 and mouse peritoneal macrophages^36^, and recovered the viability of the heterologous *hmp* deficient *Salmonella* within mouse peritoneal macrophages^14^. However, analysis of the genomes of *M. tuberculosis* and *H. pullorum* shows that *M. tuberculosis* encodes one flavohaemoglobin and two truncated haemoglobins whereas *H. pullorum* expresses one truncated and a single domain haemoglobin. Therefore, the occurrence of a different number and type of haemoglobin-like genes in the two genomes does not allow a simple comparison between the behaviour of the two bacteria when infecting macrophages.

The biochemical characterization of *H. pullorum* haemoglobins indicated that oxidised SdHb and TrHb have, respectively, a low- and a high-spin haem *b* iron. Studies done on *C. jejuni* and *Vitreoscilla sp*. strain C1 reported that the single domain haemoglobins have the haem mainly in a high-spin state, and the truncated *C. jejuni* Ctb occurs as a mixture of low- and high-spin states^37, 38^.

The two *H. pullorum* haemoglobins investigated here exhibited in the oxygenated state high reactivity towards NO, with the *H. pullorum* SdHb presenting a second order rate constant (3.6 × 10^7^ M^−1^ s^−1^, 5 °C) that is much greater than that measured for TrHb at 5 °C (3 × 10^5^ M^−1^ s^−1^) or at 20 °C (9 × 10^5^ M^−1^ s^−1^). The reactivity of *H. pullorum* TrHb with NO is comparable to that of the *M. tuberculosis* truncated haemoglobin HbO (6 × 10^5^ M^−1^ s^−1^ at 23 °C^39^), but remarkably lower than that of the *M. bovis* truncated haemoglobin HbN (7.45 × 10^8^ M^−1^ × s^−1^ at 23 °C)^15^. On the contrary, *H. pullorum* SdHb and *M. bovis* HbN seem to have a comparable reactivity towards NO (3.6 × 10^7^ M^−1^ s^−1^ at 5 °C to be compared with 7.45 × 10^8^ M^−1^ × s^−1^ at 23 °C^39^, taking into account the different temperature between the two studies).

Like in other studies^40–42^, the two *H. pullorum* haemoglobins characterized here were His-tagged and displayed unaltered properties (expected apparent molecular mass, correct heme incorporation, characteristic spectral properties and prompt reactivity towards NO in the oxy state), in line with a previous study carried out on cytoglobin showing no effect of the His-tag on the protein biochemical and functional properties^43^.

Our amino acid sequence analysis indicates that the *H. pullorum* Prx1 is a homolog of bacterial Bcps. Additionally, we observed that the expression of the *prx*1 gene remained unchanged under stress conditions and that Prx1 did not protect against hydrogen peroxide or peroxynitrite. We found that the *H. pullorum prx2* is up-regulated in peroxynitrite-treated cells and the ∆*prx2* mutant (as well as the ∆*prx1*∆*pxr2* double mutant) exhibits severe growth defects under standard microaerobic growth conditions. Likewise, the *H. pylori* strain mutated in *ahpC* (a homolog of the *H. pullorum* Prx2) could only be obtained under low oxygen conditions, being highly susceptible to the normal microaerobic growth conditions of *H. pylori* ^44^. The *H. pullorum prx3* gene is upregulated in response to hydrogen peroxide and the strain lacking the gene seemed to be not viable under standard growth conditions.

We thus speculate that the unmodified phenotype of the ∆*prx1* mutant compared to the wild type may be related to the presence of the two other peroxiredoxins, possibly compensating the absence of Prx1. This would be consistent with the peroxynitrite reactivity displayed by the peroxiredoxins Prx1, Prx2 and Prx3. Indeed, reduced Prx3 exhibited the highest reactivity toward peroxynitrite among the three *H. pullorum* peroxiredoxins, the reaction being at least one order of magnitude faster than that measured with Prx1 and Prx2. The activity of Prx3 is of the same order of magnitude of *M. tuberculosis* Tpx (homolog of Prx3), which was shown to reduce peroxynitrite with an activity approximately one log higher than the ones reported for the bacterial AhpC enzymes^26, 45^. It is also worth mentioning that in *C. jejuni* the single *bcp* mutation did not increase the sensitivity of the strain to oxidative and nitrosative stress agents, but only when harbouring a double mutation of *bcp* and *tpx* genes the strain became hypersensitive to these stresses^46^.

A distinct behaviour of the peroxiredoxins mutant strains was also observed in other organisms during macrophage infection. While *M. tuberculosis* and *H. cinaedi ahpC* provided protection from killing by macrophages^25, 47^, *Burkholderia cenocepacia bcp* was not required for survival^48^, and the *S. aureus ahpC* mutant strain did not show higher susceptibility to neutrophil-dependent killing^49^. Moreover, the *Francisella tularensis ahpC* mutant had unchanged replication in J774 cells, but impaired replication in bone marrow-derived macrophages^23^. Similarly, and consistently with our *in vitro* data, deletion of *prx1* did not lower the survival of *H. pullorum* during macrophage infection.

In conclusion, by combining physiological studies with biochemical data on five different recombinant proteins, we elucidated for the first time the nitrosative defence mechanisms of *H*. pullorum. We report that *H. pullorum* expresses a single domain and a truncated haemoglobin, the former playing the main role in the protection of *H. pullorum* against nitrosative stress conditions. Indeed, *sdHb* is induced by NO releasers, and the gene product has a much higher activity with NO than the truncated haemoglobin. In agreement, the *sdHb* mutant has a clear phenotype *in vitro* as well as in the presence of murine macrophages. Moreover, *H. pullorum* expresses three peroxiredoxins that were also shown to guard *H. pullorum* from the harmful effects of nitrosative stress, by reducing peroxynitrite with significant activities, Prx3 being the fastest one.

## Methods

### Amino acid sequence analysis of *H. pullorum* genome

Genes encoding homologs of bacterial Hbs and Prxs were searched in the genome of *H. pullorum* MIT 98-5489 (available at the gene bank, assembly accession GCA_000155495.1, project ABQU00000000.1). Protein-protein BLAST algorithm at NCBI BLAST was used for amino acid sequence similarity studies, and sequence alignments were performed with Clustal ×2.1^50^ and edited with the Genedoc software^51^.

### Bacterial strains, plasmids and growth conditions

All strains and plasmids used in this study are listed in Supplementary Table S2 (Supplementary Information). *H. pullorum* 6350-92 (CCUG 33838), isolated from a stool sample of a patient with gastroenteritis and hepatitis^52^, was used as the wild type strain. Cells were routinely cultivated at 37 °C, in a microaerobic atmosphere (6% O_2_, 7% CO_2_, 3.5% H_2_, and 83.5% N_2_) generated by the Anoxomat system (Mart Microbiology), in horse blood agar (BA) composed of Blood Agar Base no. 2 (Oxoid) with 10% (v/v) defibrinated horse blood (Probiológica), supplemented with an antibiotic-antifungal mix composed by 6.3 g/L vancomycin (Roth), 3.1 g/L trimethoprim (Sigma), 2.5 g/L amphotericin B (Roth) and, when required, 20 µg/mL kanamycin or 5 µg/mL gentamicin. Bacteria were taken as fully grown when cultured in BA plates for 5 days, with two serial passages.

Unless otherwise indicated these cells, designated as fully grown *H. pullorum*, were used as the starting material in the following assays.

*E. coli* pre-cultures were grown overnight at 37 °C and 150 rpm in LB medium, supplemented when required with kanamycin, ampicillin, and isopropyl-1-thio-β-D-galactopyranoside (IPTG).

### Quantitative real-time RT-PCR analysis

Fully grown *H. pullorum* cells were inoculated in culture flasks (Nunc) filled with 10 mL Brain Heart Infusion (BHI) broth (Oxoid) plus 10% defibrinated Fetal Calf Serum (FCS, Gibco-Invitrogen) (BHI-FCS), to an initial optical density at 600 nm (OD_600_) of 0.1–0.2. Cells were grown for 19 h, at 150 rpm in microaerobic conditions, and these cultures were used to inoculate fresh BHI medium supplemented with 0.2% (v/v) β-cyclodextrin (Sigma), (BHI-βCD), at an OD_600_ of 0.1. When cells reached an OD_600_ of ~0.5, 100 µM of the nitrosative stress generator S-nitrosogluthatione (GSNO) or 50 µM hydrogen peroxide (Panreac) was added. GSNO was freshly prepared by mixing equimolar amounts of sodium nitrite and reduced glutathione under acidic conditions (0.05 M HCl)^53^. After 30 min or 1 h incubation with hydrogen peroxide or GSNO, respectively, a mixture of ethanol:phenol (95:5 v/v) was added to stabilize the RNA and cells were collected by centrifugation (10 min, 8000 g, 4 °C). As peroxynitrite reacts quickly with BHI medium, *H. pullorum* was firstly grown as described above but using Brucella Broth (Oxoid, BB) containing 5% FCS instead of BHI-FCS and BHI-βCD, and subsequently treated with 50 µM peroxynitrite (Cayman), for 15 min.

Total RNA was then isolated with the High Pure RNA Isolation kit (Roche), and residual DNA was removed by treatment with Turbo DNA-free (Ambion). RNA was quantified in a NanoDrop spectrophotometer (Thermo Scientific), and its integrity confirmed by agarose gel electrophoresis. For each sample, 200 ng RNA was converted to cDNA using the anchored-oligodT primers and the Transcriptor High Fidelity cDNA Synthesis kit (Roche).

Quantitative real-time RT-PCR experiments were done according to the manufacturer’s instructions in a LightCycler Instrument using LightCycler FastStart DNA Master SYBER Green I Kit (Roche Applied Science). The amplification reactions were carried out with equal amounts of cDNA (300 ng) as initial template, and each reaction contained 0.5 μM of the specific primers (Supplementary Table S3), 4 mM MgCl_2_, and the hot-start PCR reaction mix from Roche Applied Science. The expression ratio of the target gene was determined relatively to a reference gene, the *H. pullorum* DNA gyrase subunit A gene (*gyrA*, HPMG_00492)^54^, that does not change expression under these conditions. At least two biological samples were analysed in duplicate.

### Construction of *H. pullorum* mutants

Nonpolar single deletion mutants of truncated haemoglobin (*trHb*), single domain haemoglobin (*sdHb*), peroxiredoxin 1 (*prx1*) and peroxiredoxin 2 (*prx2*) (∆*trHb*, ∆*sdHb*, ∆*prx1* and ∆*prx2*; Supplementary Table S2) were respectively constructed by inactivation of the *H. pullorum* HPMG_00979, HPMG_00954, HPMG_00817 and HPMG_00739 genes. Inactivation was achieved by allelic exchange after transformation of *H. pullorum* with a three-fragment assembly product that consisted of the flanking regions of the target gene and the kanamycin *aphA-3* or the gentamycin *aac(3)-IV* ^55^ cassettes. To this end, two pairs of oligonucleotides (A1/A2 and B1/B2, Supplementary Table S3) were designed to amplify a 500 bp flanking region upstream (A) and downstream (B) of each *H. pullorum* gene, containing oligonucleotides A2 and B1 a 5′-extension homologous to the flanking regions of the kanamycin or gentamycin cassettes. Each product was amplified using pUC18 carrying the cassette sequences or genomic DNA of *H. pullorum* 6350-92 as templates, the primers listed in Table S3, and Phusion polymerase (Thermo Scientific). The final DNA constructs were obtained by amplifying a three-fragment assembly product combining the cassette within the two flanking regions (A and B) of each gene with oligonucleotides A1 and B2. The generated DNA fragments were introduced into *H. pullorum* 6350-92 by electroporation^56^. Briefly, full grown *H. pullorum* cells were resuspended in 2 mL ice-cold wash buffer (272 mM sucrose, 15%, v/v, glycerol), washed 3 times (4 °C, 10000 g, 10 min), and resuspended in 1/10 volume wash buffer. A cell volume of 50 mL was electroporated with 1–2 µg of DNA in a MicroPulser (Bio Rad) at 1.80 kV, 200 V, and 25 F. Immediately after electroporation, 200 µL recovery broth (BHI, 10% glycerol, 10% FCS) was added and cells were plated on non-selective BA plates containing the antibiotic mix. After overnight incubation, cells were plated on selective BA plates with 20 µg/mL kanamycin or 5 µg/mL gentamicin. The correct mutation was confirmed by visualization of three PCR products that were amplified using the positive colonies and the following primer pairs: I) A1-B2; II) A2 and a second primer matching the beginning of the kanamycin or gentamycin cassette sequence; and III) B1 with a second primer matching the end of the kanamycin or gentamycin cassette sequence^55^ (Supplementary Table S3).

The double ∆t*rHb*∆*sdHb* mutant was constructed by cross-over between the gentamycin cassette (amplified within the upstream and downstream HPMG_00979 regions, as described above) and the HPMG_00979 gene present in the genome of the previously produced ∆*sdHb* mutant, being resistant to kanamycin as a result of the first homologous recombination event. The double ∆*prx1*∆*prx2* deletion mutant was constructed by cross-over between the kanamycin cassette (amplified within the flanking fragments of HPMG_00739) and the HPMG_00739 gene in the genome of the already produced gentamycin-resistant ∆*prx1 H. pullorum* deletion mutant.

### Complementation assays

HPMG_00979 and HPMG_00954 genes were PCR amplified with Phusion polymerase from the *H. pullorum* 6350-92 genomic DNA with the primer pairs TrHb_NdeI/TrHb_XhoI and sdHb_NdeI/sdHb_XhoI (Supplementary Table S3). DNA fragments were cloned into NdeI/XhoI-pFLAG-CTC, yielding the pFLAG-*trHb* and pFLAG-s*dHb* plasmids. Competent cells of *E. coli* K-12 ATCC 23716 wild type and *E. coli* flavohaemoglobin mutant LMS2552 (∆*hmp*)^57^ were transformed with pFLAG-CTC (empty plasmid), pFLAG-*TrHb* and pFLAG-*sdHb*. Positive transformants were selected on LA medium containing either ampicillin (100 µg/mL) and kanamycin (25 µg/mL) for *E. coli ∆hmp*, or ampicillin (100 µg/mL) for *E. coli* wild type, and confirmed by PCR using the primers N26 and C24 (Supplementary Table S3).

Overnight pre-cultures of *E. coli* wild type and ∆*hmp* carrying the empty pFLAG, pFLAG-*trHb* and the pFLAG-*sdHb* were grown in LB supplemented with the respective antibiotics and 1 mM IPTG, and used to inoculate fresh LB medium supplemented with 12 µM FeCl_3_, 1 mM aminolevulinic acid and 1 mM IPTG to an OD_600_ of 0.05. Cells were grown aerobically, for 8 h, in the absence or presence of 200 µM GSNO and monitored hourly.

### Stress sensitivity assays

For the hydrogen peroxide and GSNO sensitivity experiments, fully grown *H. pullorum* was used to inoculate 10 mL of BHI-FCS in cell culture flasks at an OD_600_ of 0.1–0.2. Cells were grown for 19 h, at 150 rpm, and these cultures were reinoculated in BHI-βCD at the indicated OD_600_ values.

For the hydrogen peroxide assays, cells were diluted in BHI-βCD to an OD_600_ of 0.1, seeded into 24-well plates, and left untreated or exposed to 1 mM or 5 mM hydrogen peroxide, and growth was monitored for 24 h.

For the GSNO assays, cells were diluted in 10 mL BHI-βCD to an OD_600_ of 0.1 and grown to an OD_600_ ~0.3. At this stage, 100 µM GSNO was added to the liquid cultures and growth was monitored for 24 h.

For the peroxynitrite assays, fully grown bacteria were inoculated in 10 mL BB plus 2.5% FCS, in 25 cm^2^ cell culture flasks, to an OD_600_ of 0.1–0.2. Cells were grown for 15 h, at 150 rpm, and these cultures were inoculated to an OD_600_ of 0.05 in 10 mL fresh medium that contained 50 µM peroxynitrite. Growth was evaluated after 2, 7 and 11 h of incubation by monitoring the OD_600_. For the sensitivity assays, at least two biological samples were analysed.

### Macrophage assays

Murine macrophages J774A.1 (ATCC TIB-67) were cultured in Dulbecco’s Modified Eagle Medium (DMEM) with high glucose, GlutaMAX™ Supplement, pyruvate (GIBCO), 10% FCS and 100 μg/mL penicillin/streptomycin (Sigma) (DMEMc), and incubated, at 37 °C, in a humidified 5% CO_2_-air controlled atmosphere.

Fully grown *H. pullorum* was plated on BA plates for 24 h, inoculated in BB containing 5% FCS at an OD_600_ of 0.1–0.2 and grown for 15 h. Immediately before the infection, *H. pullorum* cells were pelleted (10 min, 8740 g, 4 °C), resuspended in Roswell Park Memorial Institute 1640 Medium (RPMI) at an OD_600_ of 0.2, and plated for CFU counting prior to incubation in macrophages (time zero of infection).

For the infection assays, macrophages were first resuspended in RPMI with GlutaMAX™ Supplement containing 10% FCS and 100 μg/mL penicillin/streptomycin (RPMIc), and seeded overnight in 24-well plates at a concentration of 2 × 10^5^ cells/well. Macrophages were activated with 0.15 μg/mL gamma interferon (INF-γ, Sigma) and 0.5 μg/mL lipopolysaccharides (LPS, Sigma) for 5 h. Inhibition of iNOS activity was achieved by addition of 800 μM N^G^-monomethyl-L-arginine acetate salt (L-NMMA, Sigma). Prior to infection, macrophages were resuspended in RPMI GlutaMAX™ plus 10% FCS (RPMIi) and, where indicated, supplemented with the L-NMMA inhibitor.

Macrophages were infected with *H. pullorum* wild type and mutant strains, at a multiplicity of infection (MOI) of 100, for 2 and 6 h. At these time points, the supernatants were collected and the NO production was assessed from the amount of nitrite produced, using the Griess method^58^. After three washing steps with PBS, the macrophages were lysed with 2% saponin (w/v) and the bacterial content evaluated by CFU counting. Two independent biological samples were analysed in duplicate.

### Production of recombinant proteins

*H. pullorum* Hb- and Prx-encoding genes were amplified from genomic DNA by PCR using Phusion polymerase and the primers described in Supplementary Table S3, originating the following DNA fragments that contained the complete sequence of the coding region of each gene: 436 bp for HPMG_00979 (*trHb*), 563 bp for HPMG_00954 (*sdHb*), 506 bp for HPMG_00817 (*prx1*), 628 bp for HPMG_00739 (*prx2*) and 535 bp for HPMG_00529 (*prx3*). After cloning into NdeI/XhoI-digested pET28a + (Novagen) and transformation into in *E. coli* XL1 Blue, the positive recombinant plasmids were selected from kanamycin (30 μg/mL)-resistant colonies in Luria-Bertani Agar (LA) medium, and the DNA sequence integrity of all genes was confirmed by sequencing.

*E. coli* BL21 Gold (DE3) cells harbouring each of the recombinant expression plasmids were aerobically grown in LB supplemented with 30 μg/mL kanamycin, at 37 °C and 150 rpm, until an OD_600_ of 0.8 was reached; at this stage 400 μM IPTG was added and cells were incubated overnight, at 20 °C. Cells were harvested by centrifugation (10 min, 8000 g at 4 °C), resuspended in Tris-HCl 20 mM pH 7.5 (buffer A), disrupted in a French pressure cell, and centrifuged at 100,000 g, for 2 h at 4 °C.

The soluble fractions were loaded onto a charged nickel-IMAC Sepharose HP column (GE Healthcare) equilibrated with buffer A with 500 mM NaCl, eluted with approximately 500 mM imidazole, and dialysed overnight against buffer A containing the following salt concentrations: 150 mM NaCl for Prxs, or 250 mM NaCl and 20% glycerol for Hbs.

Protein purity was evaluated by SDS/PAGE and protein concentration determined by the bicinchoninic acid assay^59^ (for the globins) or using the 280 nm calculated extinction coefficient (for the peroxiredoxins).

Haem quantification in Hbs was performed by the haemochromopyridine method according to the procedure previously described^60^. The haemoglobin concentrations reported throughout the text refer to the haem-bound holoproteins.

### Spectroscopic studies

UV-visible absorption spectra of haemoglobins (10 µM) were recorded, at room temperature, in a Shimadzu UV-1700 spectrophotometer.

EPR spectra were obtained in a Bruker EMX spectrometer equipped with an Oxford Instruments continuous flow helium cryostat and were recorded at 12 K, 9.4 GHz microwave frequency, 2.0 mW microwave power and 1 mT modulation amplitude.

### Stopped-flow experiments

Stopped-flow experiments were carried out in a thermostated stopped-flow instrument (DX.17MV, Applied Photophysics) equipped with a 1-cm path length observation chamber.

The reaction of NO with *H. pullorum* haemoglobins (SdHb and TrHb) was investigated by stopped-flow multiwavelength spectrophotometry by mixing the proteins in the ferrous oxygenated state with an anaerobic solution containing NO at varied concentrations. NO stock solutions were freshly prepared before each experiment by equilibrating degassed ultra-pure water with authentic NO gas at 1 atm, yielding 2 mM NO in solution; the solution was kept on ice protected from light and diluted in anaerobic buffer to the desired NO concentration just before mixing in the stopped-flow apparatus. Prior to the experiment, the proteins were reduced with a few grains of dithionite; after removing the excess dithionite by gel filtration, the proteins were converted in the ferrous oxygenated state by dilution in air-equilibrated buffer. Formation of the ferrous oxygenated proteins was confirmed spectrophotometrically, before mixing the proteins with NO in a 1:1 (v/v) ratio in the stopped-flow apparatus. In these experiments, the stopped-flow instrument was used in the multi-wavelength mode, i.e., interfaced with a photodiode-array. To avoid light artefacts, UV light below 360 nm in the white-light incident beam was filtered off. The reactions were investigated at 5 °C or 20 °C in 50 mM potassium phosphate, 50 μM EDTA pH 7.5, by collecting absorption spectra in the 360–740 nm range as a function of time, with an acquisition time of 1 ms per spectrum.

The reaction of peroxynitrite (ONOO^−^) with the reduced proteins Prx1, Prx2 and Prx3 from *H. pullorum* was investigated by time-resolved spectroscopy, according to the ‘initial rate approach’ previously described^31, 32^. The proteins were reduced by 10 mM dithiothreitol (DTT) by incubation for 2 h at room temperature. Prior to the experiments, the excess DTT was removed and the buffer exchanged to 100 mM phosphate buffer pH = 7.0 containing 0.2 mM diethylenetriamine pentaacetic acid (DTPA) by concentration/dilution cycles. Afterwards, the proteins were gently degassed and anaerobically mixed in a 1:1 (v/v) ratio at increasing concentrations (from 5 to ~200 µM) with a solution of 40–50 µM ONOO^−^ in 10 mM NaOH. The reaction was followed at 5 °C. The initial rate of ONOO^−^ decomposition (*V*_*o*_) was obtained from the absorption decrease measured at 310 nm, using ε = 1.6 × 10^3^ M^−1^ cm^[−1 31, 32^. The kinetic traces were analysed from 4 ms on, because a small artefactual signal was invariantly observed over the very first millisecond after mixing. Given the rate law *V*_*o*_ = *k* [ONOO^−^]_0_ [Prx]_0_, the second-order rate constant (*k*) of the reaction was estimated by linear regression analysis of the dependence of the measured initial rates on protein concentration, and dividing the fitted slope by the initial ONOO^−^ concentration ([ONOO^−^]_0_). The concentration of peroxiredoxins was obtained using the following 280 nm extinction coefficients predicted by Bioinformatics Resource Portal of the Swiss Institute of Bioinformatics (http://www.expasy.org/): 24.1 mM^−1^ cm^−1^, 19.6 mM^−1^ cm^−1^ and 3.1 mM^−1^ cm^−1^ for Prx1, Prx2 and Prx3, respectively.

Kinetic data were analysed with the software MATLAB (Mathworks, South Natick, MA). When indicated, global fit analysis of spectral data was performed by singular value decomposition analysis combined with curve fitting^61^.

## Electronic supplementary material

Supplementary Information
