# Supplementary material for: Nitrosative stress defences of the enterohepatic pathogenic bacterium Helicobacter pullorum
Source: Sci Rep. 2017 Aug 30;7:9909. doi: 10.1038/s41598-017-10375-1 (PMC5577044; doi:10.1038/s41598-017-10375-1)
Supplement: Supplementary file 1 — Supplementary Information [file 41598_2017_10375_MOESM1_ESM.pdf]

Supplementary Information

Nitrosative stress defences of the enterohepatic pathogenic bacterium *Helicobacter pullorum*

Margarida R. Parente[1], Elena Forte[2], Micol Falabella[2], Ivo G. Boneca[3,4] Miguel Teixeira[1], Alessandro Giuffrè[5] and Lígia M. Saraiva[1 *]

1. Instituto de Tecnologia Química e Biológica António Xavier, NOVA, Av. da República, 2780-157, Oeiras, Portugal
2. Department of Biochemical Sciences, Sapienza University of Rome, Piazzale Aldo Moro 5, I-00185- Rome, Italy
3. Institut Pasteur, Groupe Biologie et Génétique de la Paroi Bactérienne, Département de Microbiologie, Paris, France
4. INSERM, Groupe Avenir, Paris, France
5. CNR Institute of Molecular Biology and Pathology, Piazzale Aldo Moro 5, I-00185 Rome, Italy

[*] Corresponding author:
Lígia M. Saraiva
Instituto de Tecnologia Química e Biológica NOVA
Av. da República, 2780-157, Oeiras, Portugal.
E-mail: lst@itqb.unl.pt; Phone: +351-214469328; Fax: +351-214411277

Running Title: NO defences of *H. pullorum*

Table S1. Absorption maxima and estimated extinction coefficients of *H. pullorum* globins

Globin	Protein species	λ_{max}	ε (mM^{-1} cm^{-1})
**SdHb**	deoxy (Fe^{2})	429	103.5
		551	13.2
	oxy (Fe^{2+}- O_2)	407	114.4
		543	13.2
		577	9.6
	met (Fe^{3+})	403	104.7
		501	11.0
**TrHb**	deoxy (Fe^{2})	433	131.4
		565	15.8
	oxy (Fe^{2+}- O_2)	414	120.7
		543	12.9
		573	10.1
	met (Fe^{3+})	410	127.0
		515	10.9

Table S2. Strains and plasmids used in this study

Strain or plasmid	Genotype or relevant characteristics	Source
**Strains**		
***H. pullorum* 6350-92**	Parental strain	46
Δ*trHb*	HPMG_00979 deletion mutant; Km^r	This study
Δ*sdHb*	HPMG_00954 deletion mutant; Km^r	This study
Δ*trHb*Δ*sdHb*	HPMG_00979 and HPMG_00954 deletion mutant; Km^r Gm^r	This study
Δ*prx1*	HPMG_00817 deletion mutant; Gm^r	This study
Δprx*2*	HPMG_00739 deletion mutant; Km^r	This study
Δ*prx1*Δ*prx2*	HPMG_00817 and HPMG_00739 deletion mutant; Km^r Gm^r	This study
E. coli		
K-12 ATCC 23716	Parental strain	ATCC
Δ*hmp*	LMS2552; K-12 *hmp* mutant; Km^r	51
XL1-Blue	*recA1 endA1 gyrA96 thi-1 hsdR17 supE44 relA1 lac* [F′ *proAB lacIq* ZΔM15 Tn*10*]; Tet^r	Agilent Technologies
BL21 Gold (DE3)	F^- *ompT hsdS*(r_B^- m_B^-) *dcm*$^+$ *galλ endA* Hte; Tet^r	Stratagene
**Plasmids**		
pFLAG-CTC	Vector for protein expression under the influence of the *tac* promoter; Amp^r	Sigma
pFLAG-CTC-*trHb*	pFLAG-CTC carrying the *trHb* gene subcloned into NdeI and XhoI sites; Amp^r	This study
pFLAG-CTC-*sdHb*	pFLAG-CTC carrying the *sdHb* gene subcloned into NdeI and XhoI sites; Amp^r	This study

Table S3. Primers used in this study

Primer designation	Oligonucleotide sequence (5' to 3')
**Quantitative real-time RT-PCR**	
gyrA_RT_fw	GAGGCTTATGAAACAGGGAGAGG
gyrA_RT_rv	CATAGGGGTTGATTTAAAAAGGTGA
TrHb_RT_fw	TGTGTGCCTCCCACTCTATATCG
TrHb_RT_rv	ATGGGCTTGGCGAGATTTT
SdHb_RT_fw	GAGTGTTTGCTTGTGGCGATT
SdHb_RT_rv	CCACGCCTCTAGCACTTCATC
Prx1_RT_fw	TAACCCCCAAGCACCATAAG
Prx1_RT_rv	GAGGAAGTTGGGTAGTTTTGTATTT
Prx2_RT_fw	CAACCAGCAGGGCAAACTT
Prx2_RT_rv	ACTCCGGTAAATCAAGGTGGTAT
Prx3_RT_fw	GCGAACCTGCCAAAATCACAC
Prx3_RT_rv	TAGGCGGAGCAAGTGGGAAGT
**Gene deletion**	
TrHb_A1	CTTGCATTTCCAAAATCCTAACCAT
TrHb_A2	TTATTCCTCCTAGTTAGTCAATAAAATCTCCTTATAAATAAAATGC
TrHb_B1	TACCTGGAGGGAATAATGAGCTAAAGTGGTGGTAAATTTACCTCAATCTATCC
TrHb_B2	GCGGGTATCCTGATTGTAGAGAGATTTTTTATC
SdHb_A1	CCTACAAACTTCTGTCCATCAAATCTAATAAGGGGCTG
SdHb_A2	TTATTCCTCCTAGTTAGTCAAATAAATCCTTTTGACTTCA
SdHb_B1	TACCTGGAGGGAATAATGATTGATTTGCAAAAAAACTT
SdHb_B2	GTATGAGGGAGGCTTGGCGACAATGCGTTATTCTATC
Prx1_A1	TAAAGGAGAGCAAATAGAGCA
Prx1_A2	TTATTCCTCCTAGTTAGTCACTTATATCCTTGTTAAGTTTT
Prx1_B1	TACCTGGAGGGAATAATGAAAGAATTGCAAGGCTAACT
Prx1_B2	TCTATTGTCTTTGGAGGAA
Prx2_A1	ATATCGGGGCTTTGTTCTC

Prx2_A2	TTATTCCTCCTAGTTAGTCATTTGGAGCTTTTTTTGTAAC
Prx2_B1	TACCTGGAGGGAATAATGATCGCATTCTTCTATCTTCCC
Prx2_B2	CAAATACTCTTGTGCCGTGT
**Cassette amplification**	
C1	GGAATTGTGAGCGGATAAC
C2	CCAGTCACGACGTTGTAAA
**Confirmation of**	**the mutation**
H50	CCGGTGATATTCTCATTTTAGCC
H17	TTTGACTTACTGGGGATCAAGCCTG
H121_1	CGGCTTTTCGCCATTCGTATT
H122_2	CTTCCTGCATCCGCCAGAGGC
**Gene cloning**	
TrHb_NdeI	GGAGATCATATGCAATATCAAGAAA
TrHb_XhoI	GTTCCAAAGGATAGCTCGAGGTAAA
SdHb_NdeI	TTTCATATGTTAGATATACAAAC
SdHb_XhoI	AAGATCTCGAGAAAATCAAAAA
Prx1_NdeI	CATATGGAATTGCAAATTGGAGAT
Prx1_XhoI	CAAACTCGAGAAAGAAGTGCTAAC
Prx2_NdeI	GGATTTCATATGTTAGTTAC
Prx2_XhoI	GAAGCCTCGAGCAACTTAG
Prx3_NdeI	TTAATAAGGATAAACATATGGTTACTT
Prx3_XhoI	TTCTATCTAAACTCGAGCAACAC
N26	CATCATAACGGTTCTGGCAAATATTC
C24	CTGTATCAGGCTGAAAATCTTCTC

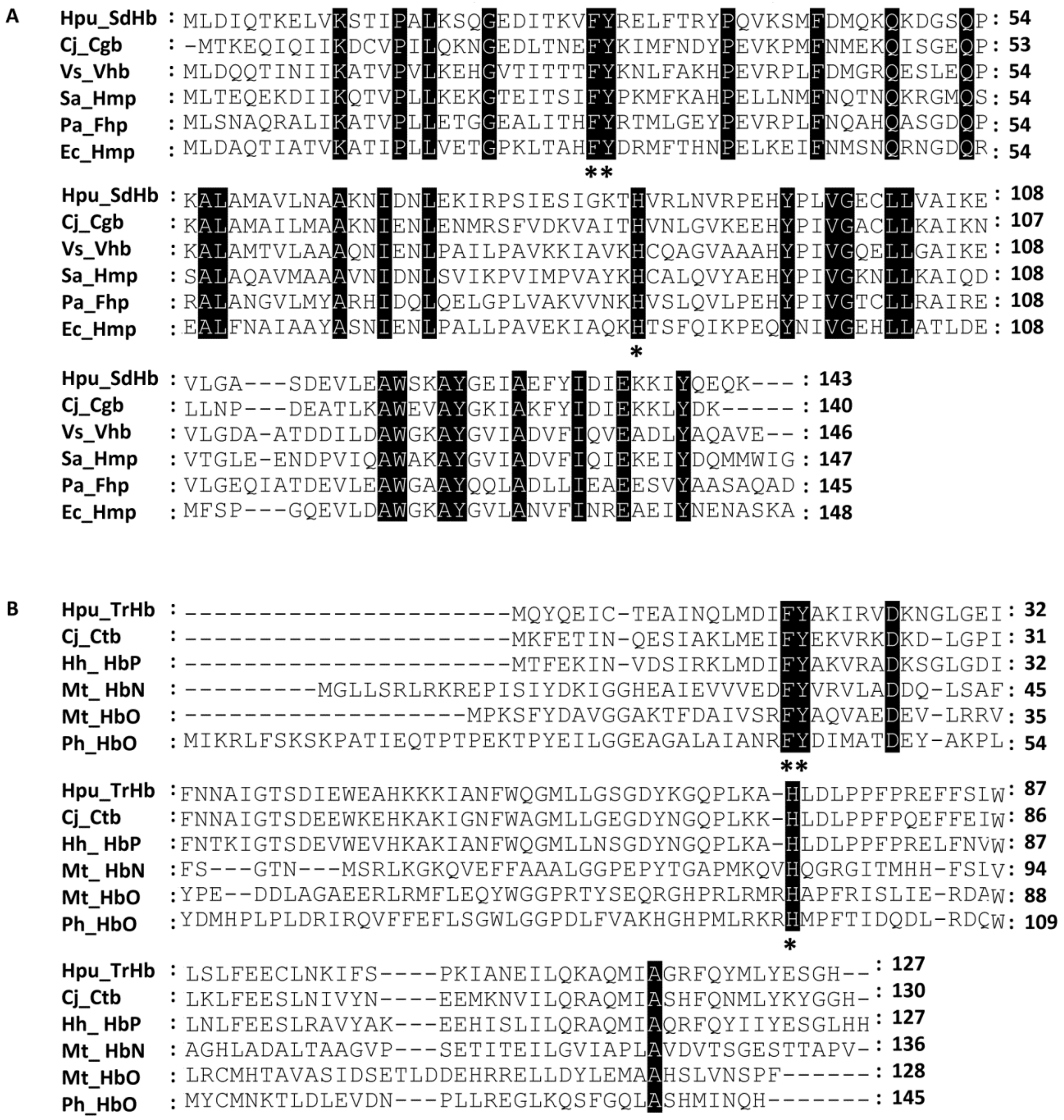

Figure S1. Amino acid sequence comparison of *H. pullorum* haemoglobins.

Amino acid sequences of *H. pullorum* SdHb (HPMG_00954) (A) and TrHb (HPMG_00979) (B) are compared with the best characterised bacterial haemoglobins. Figure 1A depicts the protein sequences of the single domain haemoglobins of *H. pullorum* (Hpu_SdHb), *C. jejuni* (Cj_Cgb) and *Vitreoscilla stercoraria* (Vs_Vhb), and the globin domain of flavohaemoglobins of *S. aureus* (Sa_Hmp), *Pseudomonas aeruginosa* (Pa_Fhp) and *E. coli* (Ec_Hmp). In Figure 1B are represented the truncated haemoglobins of *H. pullorum* (Hpu_TrHb), *C. jejuni* (Cj_Ctb), *H. hepaticus* (Hh_HbP), *M. tuberculosis* (Mt_HbN), *M. tuberculosis* (Mt_HbO), and *Pseudoalteromonas haloplanktis* (PSHAa0030 gene, Ph_HbO). Conserved amino acids residues are shaded in black and asterisk denotes the proximal histidine F8 residue that binds haem and the strongly conserved phenylalanine B9 and tyrosine B10 residues located in the haem

pocket, where tyrosine B10 stabilizes the haem ligation [12]. Alignments were done with Clustal X 2.1.

```
Hpu_Prx1  : ----------------MELQIGDKAPNFSLPNQDNAEISLQDFRGSWVVLYFYPK: 39
Hpy_Bcp   : ---------------MEKLEVGQLAPDFRLKNSDGVEISLKDLLHKKVVLYFYPK: 40
Cj_Bcp    : ----------------MSLNIGDKAPQFELLNQDGVKIALKDFIGKKVILYFYPK: 39
Hh_Bcp    : ----------------MKLQKGDKAPQFRLKNADEIEISLQDLLTKRVVVYFYPK: 39
Sa_Bcp    : -----------------MLQKGEQFPIFKLENQDGTVITNDTLKGKKAIIYFYPR: 38
Ec_Bcp    : ---------------MNPLKAGDIAPKFSLPDQDGEQVNLTDFQGQRVLVYFYPK: 40
Hpu_Prx2  : ------------MLVTKKAPNFKAPAVLADNQIVEDFELARNLGRNGAVVFFWPK: 43
Hpy_AhpC  : ------------MLVTKLAPDFKAPAVLGNNEVDEHFELSKNLGKNGAILFFWPK: 43
Cj_AhpC   : ------------MIVTKKALDFTAPAVLGNNEIVQDFNLYKNIGPKGAVVFFYPK: 43
Hh_AhpC   : ------------MLVTKPAPDFTAEAIKADGTFEDSFNLYKNIGKNGAVVFFWPK: 43
Sa_AhpC   : ------------MSLINKEILPFTAQAFDPKKDQFKEVTQEDLKGSWSVVCFYPA: 43
Ec_AhpC   : --------------MSLINTKIKPFKNQAFKNGEFIEITEKDTEGRWSVFFFYPA: 41
Hpu_Prx3  : ---MVTFKGNAVSLKGKEINVGDSAPKVELIAGDLSAKSVGGASGKFQIINVVPS: 52
Hpy_Tpx   : -MQKVTFKEETYQLEGKALKVGDKAPDVKLVNGDLQEVNLLKQGVRFQVVSALPS: 54
Cj_Tpx    : --MSIVNFKGNPVKLKGNSVEVGADAPKVNLKAKDLSVIEIGAAGKTQIILSVPS: 53
Sa_Tpx    : -MTEITFKGGPIHLKGQQINEGDFAPDFTVLDNDLNQVTLADYAGKKKLISVVPS: 54
Ec_Tpx    : MSQTVHFQGNPVTVANSIPQAGSKAQTFTLVAKDLSDVTLGQFAGKRKVLNIFPS: 55
                                                                   *

                ↓
Hpu_Prx1  : DKTPGCTQEACDFRDNLANLSGLNAVVLGVSPDSVKTHQSFIDKE-------SLN : 87
Hpy_Bcp   : DNTPGCTLEAKDFSALFSEFEKKNAVVVGISPDNAQSHQKFISQC-------SLN : 88
Cj_Bcp    : DNTPGCTTEACDFSANYDKFGGKNAVIIGISPDSVASHEKFISKF-------DLK : 87
Hh_Bcp    : DNTPGCTIEAEEFSTLLEKFEAKDTIIVGISPDSPKCHQNFINKK-------ALK : 87
Sa_Bcp    : DNTPTCTTEACDFRDNLEMFNDLDVAVYGISSDSKKKHQNFIEKH-------GLN : 86
Ec_Bcp    : AMTPGCTVQACGLRDNMDELKKAGVDVLGISTDKPEKLSRFAEKE-------LLN : 88
Hpu_Prx2  : DFTFVCPSEIIAMDHRVKAFAEKGFNVIGVSIDSDVVHFAWKNTPVNQGGIGNVQ : 98
Hpy_AhpC  : DFTFVCPTEIIAFDKRVKDFQEKGFNVIGVSIDSEQVHFAWKNTPVEKGGIGQVT : 98
Cj_AhpC   : DFTFVCPSEIIAFDKRYQEFKNRGIEVIGISGDNEFSHFAWKNTPVNQGGIGQVK : 98
Hh_AhpC   : DFTFVCPSEIIAFDKRVKDFEARGVKVIGVSIDSKEVHFAWRNVPVNQGGIGAVT : 98
Sa_AhpC   : DFSFVCPTELEDLQNQYEELQKLGVNVFSVSTDTHFVHKAWHDHS---DAISKIT : 95
Ec_AhpC   : DFTFVCPTELGDVADHYEELQKLGVDVYAVSTDTHFTHKAWHSSS---ETIAKIK : 93
Hpu_Prx3  : LDTGVCATQTRKFNEKAASLS--NAEVFVVSLDLPFAQGRFCSIE------GIQN : 99
Hpy_Tpx   : LTGSVCQ--AKHFNEQTGKLP--SVSFSVISMDLPFSQGQICGAE------GIKD : 99
Cj_Tpx    : LDTPVCATEAREFNKKVASYN--GAEVIVVSMDLPFAMGRFCSTE------GIEN : 100
Sa_Tpx    : IDTGVCDQQTRKFNSDASKE---EGIVLTISADLPFAQKRWCASA------GLDN : 100
Ec_Tpx    : IDTGVCAASVRKFNQLATEID--NTVVLCISADLPFAQSRFCGAE------GLNN : 102
             *
```

Figure S2. Amino acid sequence comparison of *H. pullorum* peroxiredoxins.

Amino acid sequences of *H. pullorum* HPMG_00817, HPMG_00739, and HPMG_ 00529 gene products are compared with the best studied bacterial peroxiredoxins. Protein sequences: *H. pullorum* (Hpu_Prx1), *H. pylori* (Hpy_Bcp), *C. jejuni* (Cj_Bcp), *H. hepaticus* (Hh_Bcp), *S. aureus* (Sa_Bcp) and *E. coli* (Ec_Bcp) peroxiredoxins belonging to Bcp/PrxQ subfamily; *H. pullorum* (Hpu_Prx2), *H. pylori* (Hpy_AhpC), *C. jejuni* (Cj_AhpC), *H. hepaticus* (Hh_AhpC), *S. aureus* (Sa_AhpC), and *E. coli* (Ec_AhpC) belonging to AhpC/Pxr1 subfamily; and *H. pullorum* (Hpu_Prx3), *H. pylori* (Hpy_Tpx), *C. jejuni* (Cj_Tpx), *S. aureus* (Sa_Tpx) and *E. coli* (Ec_Tpx) peroxiredoxins belonging to Tpx subfamily. Conserved amino acids residues are shaded in black. The potential peroxidatic cysteine (C_P) residue is marked with an arrow, the potential resolving cysteine (C_R) is shaded in dark grey, and the non-conserved cysteines in light grey.

The conserved proline present at the catalytic site and the distal conserved arginine are marked with an asterisk. Alignments were done with Clustal X2.1.

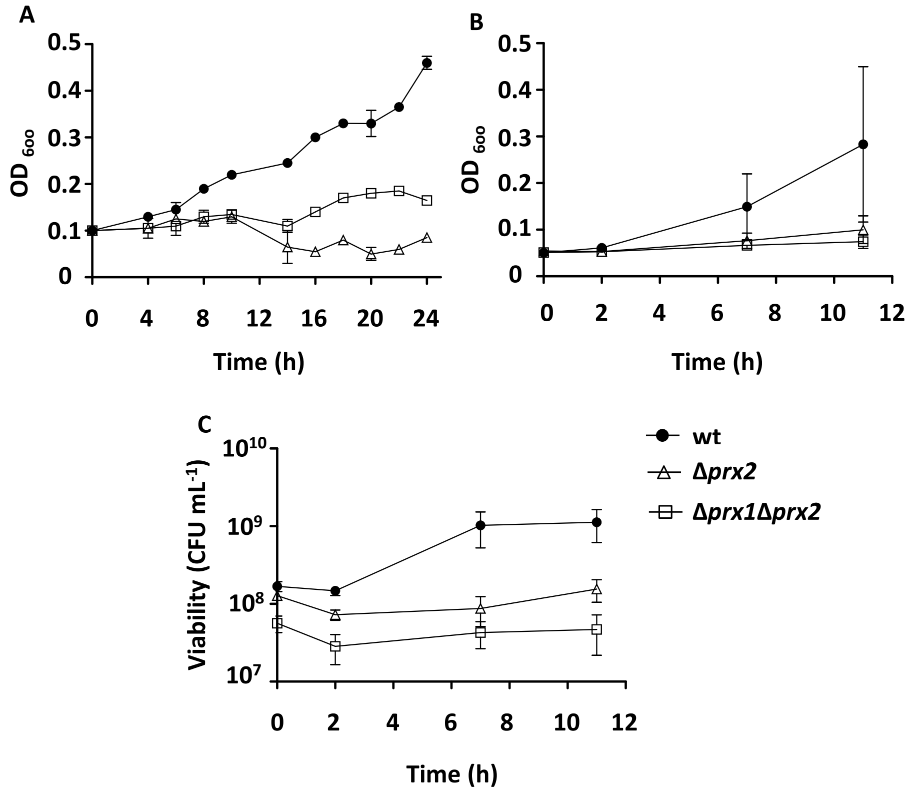

Figure S3. Viability of *H. pullorum* Δ*prx2* and Δ*prx1*Δ*prx2* mutants.

Growth curve of *H. pullorum* wild type (filled circles), Δ*prx2* mutant (open triangles) and Δ*prx1*Δ*prx2* double mutant (open squares) in BHI-βCD (A) and in BHI-βCD complemented with 2.5% FCS (B). In C is depicted the viability of the strains (as indicated in A and B) cultured in BHI-βCD complemented with 2.5% FCS. Data (mean ± standard error) acquired in two independent experiments done in duplicate.

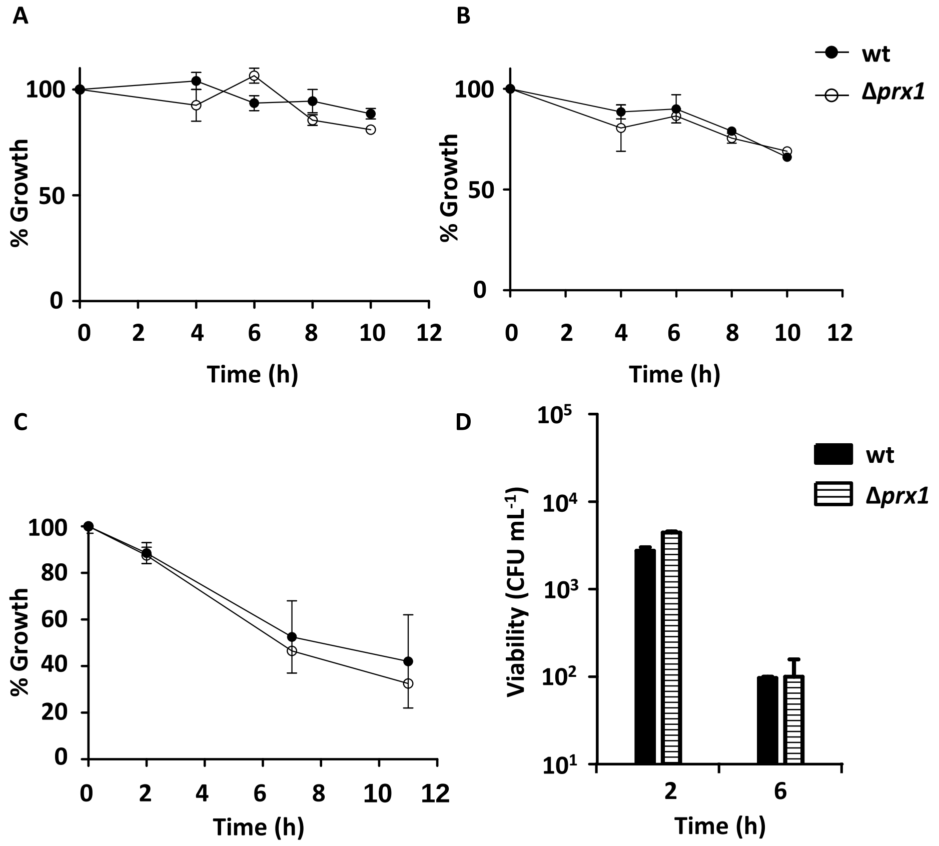

Figure S4. Effect of oxidative and nitrosative stress on the growth of *H. pullorum* wild type and Δ*prx1* mutant strains.

Growth of *H. pullorum* wild type (filled circles) and Δ*prx1* mutant (open circles) in BHI-βCD medium in the presence of 1 mM (A) or 5 mM (B) hydrogen peroxide, and in BB complemented with 2.5% FCS in the presence of 50 µM peroxynitrite (C). Activated macrophages were infected with *H. pullorum* wild type (black bar) or Δ*prx1* mutant (striped bar). Viable counts were determined after 2 and 6 h of infection (D). Data (mean ± standard error) acquired in two independent experiments.

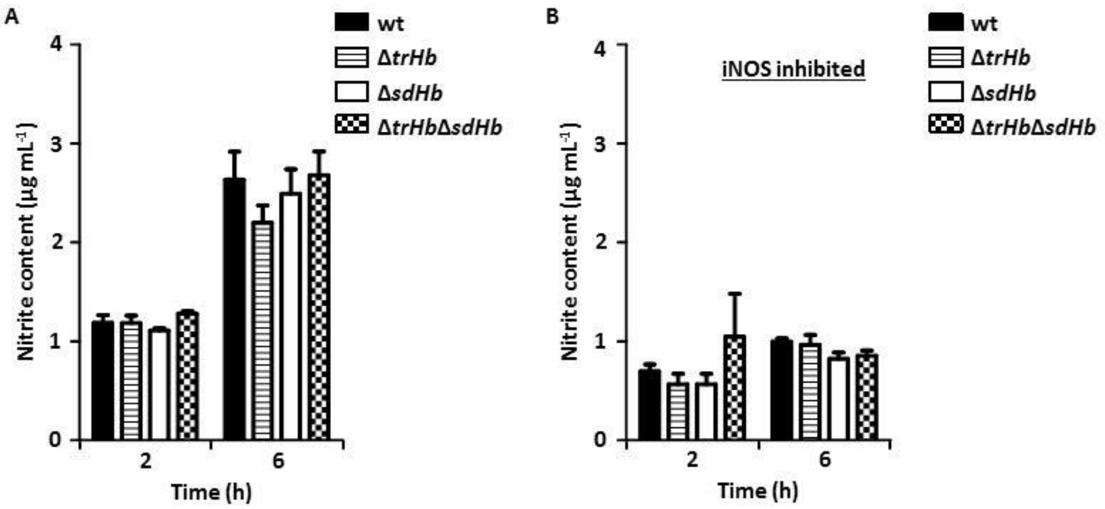

Figure S5. Nitrite content produced by macrophages infected with *H. pullorum* wild type and haemoglobin mutant strains.

NO produced by macrophages J774A.1 was evaluated in the form of nitrite in (A) activated and (B) iNOS inhibited macrophages. Macrophages were infected with *H. pullorum* wild type (black bar), Δ*trHb* (striped bar), Δ*sdHb* (white bar) and Δ*trHb*Δ*sdHb* (chess bar), at a MOI of 100. Nitrite content was determined after 2 h and 6 h of infection. At least two independent experiments were analysed in duplicate. Data represent the mean ± standard error.

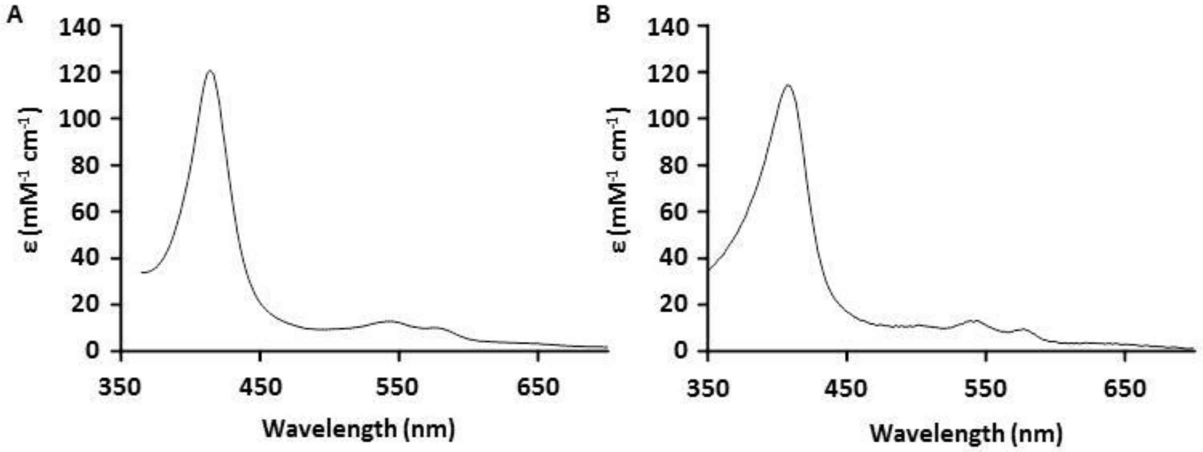

Figure S6. UV-visible spectra of *H. pullorum* ferrous oxy-TrHb (A) and ferrous oxy-SdHb (B).
